# Atypical Psoriatic Arthritis Presenting as Presumed Septic Monoarticular Arthritis

**DOI:** 10.7759/cureus.10470

**Published:** 2020-09-15

**Authors:** Zachary Sanford, Ralph W Webb

**Affiliations:** 1 Rheumatology, Marshall University Joan C. Edwards School of Medicine, Huntington, USA

**Keywords:** psoriatic arthritis, septic arthritis, candida parapsilosis

## Abstract

The authors present a case of monoarticular psoriatic arthritis affecting the knee of a woman with no previously identified manifestations of psoriasis. At time of symptom onset, joint aspirates were initially sterile with predominantly neutrophilic leukocytosis; however, repeat aspiration and culture was positive for Candida parapsilosis. Despite appropriate antifungal treatment, symptoms continued to worsen and other large joint manifestations including the contralateral elbow raised suspicions for alternative etiology. Ultimately new onset skin excoriations on the palms suggested psoriatic arthritis, which was subsequently treated with immunosuppressive therapy.

## Introduction

Psoriatic arthritis (PsA) is an erosive and painful synovitis afflicting up to 30% of patients diagnosed with psoriasis [1,2]. A diagnosis of exclusion, PsA is a seronegative arthritis often affecting multiple large and small joints in an asymmetrical fashion, presenting with dactylitis, enthesitis, or inflammation of the tendinous or ligamentous insertions into the bone, spondylitis, and nail manifestations [3]. Peripheral or axial joints are subject to a variable course of bone resorption and proliferation, potentially resulting in gross deformity [4]. Acute phase reactants, such as erythrocyte sedimentation rate (ESR) and C-reactive protein (CRP), are often elevated but are not unique to the disease. While synovial fluid analysis typically yields nonspecific sterile leukocytosis with neutrophil predominance, we describe a case of PsA masked by superimposed synovial fluid leukocytosis and fungal isolate.

## Case presentation

A 48-year-old Caucasian female presented to a local urgent care center with two-month history of abrupt onset of left knee pain, stiffness, and swelling without antecedent trauma or precipitating events. Past medical history was significant for type II diabetes mellitus, hypothyroidism, eczema, and gastroesophageal reflux disease. Home medications consisted of levothyroxine and sitagliptin/metformin. The patient had been using over-the-counter azole antifungal cream for what she stated was tinea corporis.

The patient complained of subjective joint pain, swelling, and instability, and pain was worse with ambulation. Her symptoms had progressed to the point that the she relied on crutches to assist with ambulation. Pertinent examination findings included left knee effusion and crepitus as well as mildly dry skin and scaling in the palms and soles. Standard radiography at presentation was noncontributory, and the patient was presumed to have possibly torn her medial meniscus. The patient underwent joint aspiration which yielded 13,800 white blood cells (WBCs) with 93% neutrophils, few red blood cells (RBCs), no crystals, and negative aerobic culture. Corticosteroid injection was administered with minimal relief of pain.

Two weeks later, the patient presented to a regional emergency department with continuing pain and swelling in the left knee. Effusion was again noted, and the patient underwent repeat arthrocentesis. Synovial fluid at this point showed 52,470 WBCs with 87% neutrophils, 114,000 RBCs, and negative for crystals and gram stain. Anaerobic bacterial cultures were negative at five days of incubation. Serologies obtained at that time included negative rheumatoid factor, ESR 53 mm/hr (reference range 0-20), and CRP 3.59 mg/dL (reference range 0.0-0.3).

Despite the negative gram stain, the patient was presumed to have septic arthritis of the knee due to the elevated WBCs. Arthroscopic examination revealed extremely purulent material and synovitis. Biopsies demonstrated nonspecific, acute fibrinoinflammatory exudate, granulation tissue, fibrinoid degeneration, and some degree of chronic inflammation. Bacterial cultures were negative at five days, and the patient tested negative for Chlamydia trachomatis, Neisseria gonorrhoeae, and Lyme antibody. A peripherally inserted central catheter was established, and the patient began six weeks of ceftriaxone and linezolid for possible culture-negative septic arthritis.

Late cultures at one-week incubation revealed growth of Candida parapsilosis. Antifungal treatment with fluconazole was added to the antibiotic regimen, beginning with a loading dose of 800 mg followed by six weeks of 400 mg daily. After completion of two weeks of antifungal therapy, ESR decreased to 31 mm/hr, and the patient reported subjective improvement in joint stiffness. However, two weeks after completion of the full course of antifungal therapy, the left knee still appeared swollen and inflamed, and a second course of fluconazole was prescribed.

Painful edema persisted in the left knee later accompanied by the spontaneous development of swelling in the right elbow. At this time, a third synovial aspiration of the left knee yielded 68,000 WBCs with 92% neutrophils, 365,000 RBCs, negative crystals, and negative gram stain. Cultures of synovial fluid and blood remained negative. Repeat joint aspiration one week later showed persistent elevation of white blood cells at 62,000. Complete blood count showed a WBC count of 12,900 with 87% neutrophils, and ESR remained modestly elevated at 35 mm/hr.

The patient then underwent a second left knee arthroscopy with synovectomy, as well as right elbow open arthrotomy. Synovial biopsies were obtained from the suprapatellar pouch in the left knee and of the capsular lining of the right elbow. Pathology only demonstrated synovitis and fibrinous exudate. Congo red stains on the synovial biopsies for exclusion of amyloid were negative.

At this time, the eczematous eruptions on the palms and soles bilaterally were identified as palmoplantar psoriasis. Based on this finding, and considering prior negative bacterial cultures and lack of response to antibiotic therapy, the patient was presumed to have oligoarticular PsA as opposed to culture negative septic arthritis.

The patient initially started therapy with sulfasalazine 1,000 mg twice daily and a tapering dose of oral prednisone for 12 days. She felt relief initially, but her symptoms recurred after the corticosteroid therapy was completed. She was then switched from sulfasalazine to methotrexate, an anti-tumor necrosis factor (TNF) agent, and the dose was escalated over several weeks to 25 mg weekly. The patient had a variably successful response for several months; however, over time she continued to experience frequent acute flares of joint swelling and pain in the left knee, both elbows, and the left wrist. She required several courses of steroid therapy to help alleviate her symptoms. Adjunct therapy with anti-TNF agent adalimumab 40 mg s.c. every other week was added to her methotrexate regimen, and the dose was increased to a weekly schedule due to incomplete response. At this point, the patient developed injection site reactions and anti-TNF therapy was changed to etanercept. Etanercept was tolerated, but it did not seem to be effective. Anti-TNF therapy was then discontinued, and treatment with apremilast, a phosphodiesterase 4 (PDE4) inhibitor, was added to methotrexate. This combination was effective for several months; however, the patient again experienced episodes of joint pain and swelling in various sites, and she also developed a normocytic anemia. Methotrexate and apremilast were discontinued at this time, and the patient began therapy with anti-interleukin 12 (anti-IL-12) and anti-interleukin-23 (anti-IL-23) agent ustekinumab. At this time, the patient is doing well with both joint and skin symptom control, and her anemia normalized.

## Discussion

The patient appears to have PsA presenting relatively abruptly in the left knee. While she had no known antecedent psoriatic skin involvement, her palmar and plantar dermatitis that was previously attributed to eczema probably represented atypical psoriasiform lesions. Although less common than presentation after psoriatic skin lesions, the manifestation of arthritic symptoms before psoriatic skin involvement is present in as many as 15% of PsA cases [5].

The isolation of C. parapsilosis from the synovial fluid later in the patient’s clinical course is also of note insofar as this is not the most common fungal pathogen identified in immunocompetent patients. However, recent studies have suggest that C. parapsilosis and other non-albicans Candida (NAC) species may be as common or perhaps even more common than C. albicans in invasive mycoses [6,7]. It is conceivable that the excoriations on the patient’s hands exposed the vasculature to cutaneous flora, the composition of which may have been altered by topical antifungal creams such that C. parapsilosis may have gained a preferential survival advantage over C. albicans. Given the lack of response to a protracted course of antifungal therapy, the C. parapsilosis isolate may have represented a contaminant or colonization.

## Conclusions

This case illustrates the inherent difficulties in diagnosing subclinical PsA in the early stages of disease manifestation. Patients suffering from autoimmune disease may present with signs and symptoms that mimic infectious etiologies, proving challenging to the clinician in isolating a single causative etiology. We describe a case of PsA masked by superimposed synovial fluid leukocytosis and fungal isolate discovered on thorough microbiological workup at odds with expected nonspecific sterile leukocytosis with neutrophil predominance often encountered on synovial fluid analysis. This should give pause to clinicians during their investigative phase of testing; providers may be less familiar with manifestations of colonization or infection by NAC such as C. parapsilosis, prompting escalation of antifungal regimens or referral to tertiary care centers when differential diagnoses may benefit from broadening by including formal rheumatological evaluation. Still, PsA continues to serve as a diagnosis of exclusion without any clearly defined clinical markers for testing and, while representing a challenge to diagnosticians and patients alike, remains a moving target for prompt and correct identification.
